# Male Breast Cancer: Bridging the Gaps of Neglect—A Global Call for Equitable Care and Inclusion

**DOI:** 10.1002/hsr2.72687

**Published:** 2026-07-08

**Authors:** Shani Kondo Omari

**Affiliations:** ^1^ Department of Research University of Dar es Salaam(UDSM) – Mbeya College of Health and Allied Sciences (MCHAS) Mbeya Tanzania

**Keywords:** awareness and diagnosis, clinical inclusion, health equity, male breast cancer, psychosocial support

## Abstract

**Background and Aims:**

Male breast cancer (MBC) is a rare but increasingly recognized malignancy that remains underrepresented in research, policy, and clinical practice. Persistent misconceptions that breast cancer is exclusively female disease contribute to delayed presentation, advanced‐stage diagnosis, and poorer outcomes in men. This article examines global disparities affecting awareness, diagnosis, treatment, research inclusion, and psychosocial care, and proposes strategic actions to advance equitable and inclusive oncology.

**Methods:**

This article is based on a targeted review of peer‐reviewed studies, epidemiologic analyses, clinical reports, and guideline documents addressing the epidemiology, biology, diagnosis, management, genetics, and psychosocial impact of MBC. Literature was identified through structured searches of academic databases including PubMed and scholarly and authoritative institutional sources. Findings were synthesized thematically to identify gaps, systemic barriers, and priority interventions.

**Results:**

MBC accounts for approximately 0.5%–1% of all breast cancer cases globally, with evidence suggesting rising incidence and late‐stage detection. Limited public and professional awareness contributes to diagnostic delays, while absence of routine screening and male‐specific protocols further complicates early identification. Clinical management is commonly extrapolated from female breast cancer trials despite biological and hormonal differences that may influence treatment response and toxicity. Men also experience psychosocial burdens, including stigma, isolation, threats to identity, and lack of tailored support services. Pathogenic variants such as BRCA1/2, CHEK2, PALB2 are relatively prevalent among affected men, yet genetic counseling and cascade testing remain underutilized. Together, these factors create a persistent “silent burden” and contribute to suboptimal outcomes.

**Conclusion:**

Addressing inequities in MBC requires coordinated global action, including sex‐disaggregated data systems, inclusive clinical research, male‐specific diagnostic and treatment guidance, targeted awareness initiatives, and integrated psychosocial and genetic services. Recognizing MBC within national and global cancer agendas is essential to improving early detection, optimizing treatment outcomes, and ensuring equitable, evidence‐based care for all individuals affected by breast cancer.

## 1 Introduction: The Unseen Face of a Global Challenge

Breast cancer is one of the most significant global health challenges, affecting millions of lives each year. However, the dominant narrative surrounding the disease focuses primarily on women. This has shaped a deeply rooted belief in both society and medicine that unintentionally leaves male breast cancer (MBC) overlooked [1]. Historically, this exclusive focus on female patients developed because of the large difference in incidence rates, together with strong and essential advocacy by women's health movements. While this framing has been critical for improving care for the most common cancer among females, it has also unintentionally obscured the male experience of the disease.

Although MBC represents a small proportion of cases, it accounts for approximately 0.5% to 1% of all breast cancer cases and is concerning because its incidence is gradually increasing worldwide [2] [3, 4]. This rise, combined with frequent late diagnosis, contributes to higher morbidity and mortality in men compared with stage‐matched female breast cancer (FBC) patients [5]. In 2021, an estimated 38,827 new cases and 13,274 deaths were attributed to MBC worldwide, with age‐standardized incidence and mortality rates rising in many regions, including Eastern Sub‐Saharan Africa. Men are typically older at diagnosis, and they often present at more advanced stages than women, contributing to survival rates that differ from those in female breast cancer. Longitudinal analyses indicate 5‐year survival rates around 77.6% for men, with stage at diagnosis and age strongly influencing outcomes. These epidemiologic trends underscore the need for improved awareness, early detection, and sex‐specific clinical strategies [6].

Despite this growing yet under recognized burden, MBC remains under researched, underdiagnosed, and undertreated globally.

Within the broader global health landscape, where resource allocation, policy priorities, and research attention are shaped by perceived disease burden and public awareness, the marginalization of MBC is not simply an oversight [7]. Rather, it reflects a systemic gap that conflicts with the principles of universal health coverage and health equity championed by organizations such as the World Health Organization and embedded within the sustainable development goals (SDGs). The consequences extend beyond statistics, including physical and psychological suffering, financial strain on families, and reduced quality of life for affected men. These burdens are particularly pronounced among socioeconomically disadvantaged men, those living in rural or resource‐limited settings, and marginalized populations in low‐ and middle‐income countries (LMICs), where access to healthcare, specialized oncology services, and public awareness campaigns is limited [8, 9].

Men are particularly vulnerable to this neglect because prevailing gender norms can stigmatize male‐associated health conditions and limit awareness of breast health [7]. This article highlights the inequities and gaps affecting every stage of MBC care, from public awareness and diagnostic pathways to treatment protocols and psychosocial support. By examining how the prevailing female‐centric narrative contributes to systemic disparities, we present an urgent, evidence‐informed call for coordinated global action. Such efforts are necessary to ensure equitable care and meaningful inclusion of MBC within broader health agendas. Ultimately, the goal is to promote a more inclusive future in which the silent burden of male breast cancer is fully recognized, addressed, and integrated into comprehensive cancer care for all.

### 1.1 The Silent Burden: Multifaceted Challenges and Critical Gaps in Male Breast Cancer Care

The journey for men affected by breast cancer is fraught with systemic hurdles that contribute to its “silent burden”. These challenges span from the fundamental lack of awareness to significant disparities in clinical management and psychosocial support.

### 1.2 Awareness Gaps and Diagnostic Drag

One of the greatest problems facing male breast cancer patients is the widespread lack of awareness that men can develop the disease [10, 11]. This widespread lack of knowledge is a major barrier to early detection. When men find a suspicious lump or notice changes in their nipple, they often brush it off as something minor such as pulled muscle, a fatty lump, or just something harmless [12]. As a result, they may delay seeking medical attention, which can postpone diagnosis. Consequently, men are typically diagnosed at more advanced stages than women [12]. A painless lump that might immediately prompt concern in women is often overlooked in men, allowing the disease to progress unnoticed [13].

This issue is further reinforced by the persistent belief that breast cancer is exclusively a woman's disease. This idea can create a deep sense of shame and embarrassment. Men may feel reluctant to discuss breast changes or seek medical advice, which can increase isolation [14]. Such cultural barriers reinforce stigma, discourage timely care‐seeking, and promote self‐silencing. These psychosocial barriers are particularly pronounced among men in marginalized populations or resource‐limited settings, where male‐specific public health messaging and support networks are scarce, compounding the risk of delayed diagnosis [15, 16]. While public health campaigns for FBC are ubiquitous, male‐specific initiatives are virtually nonexistent, further entrenching this dangerous misconception [15]. Even within the medical community, particularly in primary care, a lower index of suspicion for MBC can lead to misdiagnoses or prolonged diagnostic pathways, reinforcing the cycle of delayed detection [16].

The diagnostic journey itself can be complex. Unlike women, men do not undergo routine mammographic screening, meaning that MBC is almost invariably detected symptomatically [17]. When a man presents with a breast mass, the diagnostic workup often mirrors that for women, involving mammography, ultrasound, and biopsy. However, interpreting male breast imaging can be challenging because of reduced glandular tissue and structural differences [18]. Radiologists, who encounter fewer male cases, may have a higher threshold for suspicion or find it harder to distinguish benign from malignant lesions [19]. Furthermore, the small amount of breast tissue can make image acquisition difficult and increase the risk of sampling errors during biopsy. The absence of clear, widely disseminated diagnostic algorithms specifically tailored for MBC may contribute to delays and highlights the need for male‐specific protocols [20]. Integrating sex‐disaggregated data into national cancer registries and implementing male‐specific diagnostic guidelines can help address these gaps and improve early detection in both high‐income and resource‐limited settings.

### 1.3 Clinical Marginalization and Exclusion

The most critical and ethically questionable gap in MBC management lies in the wholesale extrapolation of treatment protocols from FBC clinical trials. Historically, and even currently, men are largely excluded from breast cancer clinical trials because of the lower incidence of this disease in males [21]. Consequently, therapeutic decisions for MBC ranging from surgical approaches and radiation therapy to systemic treatments such as chemotherapy, hormone therapy, and targeted therapies are based almost exclusively on evidence derived from female patient populations [22].

This practice is problematic for several reasons. First, despite being the same cancer type, there are subtle yet significant biological and molecular differences between MBC and FBC, including varying expression profiles of hormone receptors with consistently higher rates of estrogen receptor (ER) positivity in MBC, HER2 status, and other genetic characteristics [23]. Treating these distinct biologies identically assumes homogeneity that may not exist and could lead to suboptimal outcomes. For example, the efficacy and long‐term side effects of endocrine therapies might differ owing to distinct hormonal environments [24]. Second, the pharmacokinetic and pharmacodynamic profiles of drugs can vary between sexes and are influenced by factors such as body composition, the hormonal milieu, and liver metabolism. Administering female‐derived dosages to men without specific validation runs the risk of suboptimal efficacy or increased toxicity [25]. Overall, exclusion from research limits understanding of optimal MBC care and slows the development of tailored therapies for men.

### 1.4 Unique Psychosocial Burden and Overlooked Genetic Imperative

Beyond these clinical challenges, men with breast cancer face a profound and often unique psychosocial burden. The diagnosis itself, may challenge masculine identity leading to feelings of emasculation, shame, and intense isolation [26]. Many men feel like anomalies, struggling to find relatable experiences or support from conventional breast cancer support groups, which are predominantly female‐centric [27]. This isolation is intensified by societal perceptions and limited public discussion of MBC, which can leave men feeling marginalized even within cancer communities [28]. A 2024 mixed‐methods systematic review found that men experience stigma and unmet supportive care needs following diagnosis, often reporting that their symptoms and psychosocial concerns are deprioritized compared with women's needs. Social constructions of breast cancer as a ‘female disease’ contribute to embarrassment and threats to masculine identity, discouraging men from disclosing symptoms or seeking care. In addition, broader sociocultural norms related to masculinity have been linked to delays in help‐seeking and lower engagement with health services, reflecting deeply rooted barriers that hinder timely diagnosis and supportive care [15]. The impact extends deeply into areas such as body image, sexuality, and relationships, with treatments such as mastectomy often causing significant psychological distress, far beyond the physical scar [28, 29]. Medical surveys confirm that while clinical anxiety and depression rates are low, nearly a quarter of men experience traumatic stress symptoms related to their diagnosis strongly linked to body image disruption [28] [30].

Furthermore, men diagnosed with breast cancer are significantly more likely to carry germline genetic mutations, such as those in *BRCA1/2*, *CHEK2*, and *PALB2*, than women with breast cancer are [23]. Despite this, genetic counseling and testing remain underutilized, limiting opportunities for cascade screening of at‐risk relatives and for guiding personalized treatment strategies [1]. Healthcare providers, often untrained in the specific psychosocial and genetic counseling needs of men with breast cancer, may inadvertently overlook these critical aspects of holistic care, further disadvantaging these patients [21].

### 1.5 A Global Call to Action: Strategies for Equitable Care and Strategic Inclusion

Male breast cancer is should not be dismissed as a statistical anomaly; it is a critical blind spot in global health and oncology that demands urgent attention. Breaking the cycle of low awareness, delayed diagnosis, extrapolated treatments, and significant psychosocial distress requires a multifaceted and globally coordinated response to advance equitable care and inclusion. Priority action is required in the following areas:

### 1.6 Integrating MBC Into Global Health Agendas

International bodies such as the World Health Organization (WHO) and national governments must explicitly include MBC in their cancer control strategies, data collection, and resource allocation frameworks [6]. Practical mechanisms include mandating sex‐disaggregated breast cancer data in national and international cancer registries, incorporating MBC modules into continuing medical education for healthcare providers, and establishing dedicated funding streams for male‐specific breast cancer research [31]. Such integration ensures that MBC is explicitly addressed within broader noncommunicable disease (NCD) initiatives, including those aligned with SDG 3, improving burden estimation, guiding resource allocation, and strengthening evidence‐informed policy development. Programmatic data from registries such as the U.S. *PDQ® Male Breast Cancer Treatment Registry* demonstrate how male case capture supports evidence generation and guideline development [32].

### 1.7 Enhanced Awareness and Education Initiatives

Public health campaigns should adopt gender‐inclusive messaging, drawing lessons from successful initiatives such as female breast cancer awareness campaigns. HIV prevention programs and male‐focused prostate cancer campaigns. For example, the Movember Foundation's global campaigns have successfully mobilized millions of men worldwide to engage with male health issues, including early detection and care‐seeking for cancer and other conditions, raising awareness through branded activities and advocacy. Operational approaches include collaborating with local community health workers, leveraging male champions or influencers, integrating MBC awareness into school and workplace health programs, and monitoring campaign reach and effectiveness using pre‐ and post‐intervention surveys [15].

Campaigns should communicate clearly, including in local languages that men can develop breast cancer and should be designed to be culturally sensitive and targeted. These initiatives should educate men, their families, and the public that breast cancer can affect anyone regardless of sex.

Multiple communication channels should be used, including social media, community radio, local health clinics, male advocates, and sporting events to broaden reach and improve awareness, particularly in settings where stigma delays care seeking

Continous professional development programs for primary care physicians, nurses, and oncology specialists are also essential. These programs can raise diagnostic suspicion, improve early recognition, and strengthen referral pathways for men.

## 2 Equitable Research Inclusion, Research Prioritization and Investment

Clinical trials should include men unless a clear biological rationale justifies exclusion. In some cases, dedicated MBC trials may also be appropriate [32]. Mechanisms to operationalize this include creating incentives for funders to prioritize MBC‐inclusive trial designs, establishing international MBC patient registries to facilitate recruitment, and setting minimum quotas for male participation in multicenter breast cancer studies.

Expanding research investment in MBC biology including molecular subtypes, genetic predispositions, and potential male‐specific biomarkers will generate sex‐specific efficacy and safety data, supporting tailored treatment strategies. Comprehensive registries and biobanks will facilitate collaborative research and provide robust data for evidence‐based guideline development.

### 2.1 Development of Male‐Specific Clinical Guidelines

Based on emerging evidence and recognition of biological differences, clinical guidance should be adapted for men. This includes diagnostic approaches, standardized imaging interpretation criteria, surgical considerations, and endocrine treatment strategies. Guidelines should be evidence‐based, informed by male‐specific data where available, and adaptable across resource settings rather than relying solely on extrapolation from female cohorts [32].

### 2.2 Building Comprehensive, Gender‐Sensitive Support Systems

Health systems should develop resources and support networks that address the psychosocial needs of men with breast cancer. These may include male‐focused counseling services and campigns such as Movember campaing online forums, peer support groups, survivor narratives, and patient navigation programs. Such initiatives can strengthen community, reduce stigma, and support coping throughout the cancer trajectory.

Healthcare providers should also receive training to address psychosocial and sexual health concerns sensitively, including body‐image and emotional well‐being.

### 2.3 Genetic Support Structures

Genetic testing should be routinely offered to men diagnosed with MBC, alongside cascade screening for at‐risk family members. Integrating genetic counseling into standard care pathways can support personalized treatment planning and family risk assessment.

## 3 Conclusion: Toward a More Inclusive and Just Future in Oncology

The under recognized burden of male breast cancer highlights a significant inequity in global oncology. It reflects structural gaps that have historically limited attention to male patients.

By acknowledging and addressing gaps in awareness, diagnosis, research inclusion, and treatment, and by ensuring that men are systematically included in health agendas, healthcare systems can move toward more equitable and effective breast cancer care. Addressing these disparities is both a scientific priority and a matter of health equity. Strengthening recognition and inclusion can help ensure that all individuals affected by breast cancer receive timely, evidence‐based, and compassionate care.

## Author Contributions

**Shani Kondo Omari:** conceptualization, writing – original draft, validation, writing – review and editing, project administration, supervision, data curation.

## Funding

The author has nothing to report.

## Ethics Statement

This article does not involve human participants, animals, or primary data collection. Therefore, ethical approval was not required.

## Conflicts of Interest

The author declares no conflicts of interest.

## Transparency Statement

Shani Kondo Omari affirms that this manuscript is an honest, accurate, and transparent account of the study being reported; that no important aspects of the study have been omitted; and that any discrepancies from the study as planned (and, if relevant, registered) have been explained.

## Data Availability

No new data were generated or analyzed in support of this manuscript. Data sharing not applicable to this article as no datasets were generated or analysed during the current study.
